# Reusable, Stable, Efficient and Multifunctional Superhydrophobic and Oleophilic Polyurethane Sponge for Oil–Water Separation Prepared Using Discarded Composite Insulator

**DOI:** 10.3390/ma16186320

**Published:** 2023-09-21

**Authors:** Meiyun Zhao, Yuanyuan Shang, Yufan Xiong, Xiaolong Zhang

**Affiliations:** 1 Hubei Key Laboratory of Hydroelectric Machinery Design & Maintenace, China Three Gorges University, Yichang 443000, China; zhaomeiyun@ctgu.edu.cn (M.Z.);; 2 College of Mechanical & Power Engineering, China Three Gorges University, Yichang 443000, China

**Keywords:** oil–water separation, absorption, separation efficiency, sponge

## Abstract

Oil spills and chemical leakages are a serious source of pollution in oceans and rivers, and have attracted worldwide attention. Many scientists are currently engaged in the development of oil–water separation technology. In this study, the umbrella skirt of a discarded silicone rubber insulator was utilized as feedstock, and polydimethylsiloxane (PDMS) was employed to immobilize the prepared powder (FXBW) onto a polyurethane (PU) sponge skeleton. Without any modifications using chemical reagents, a novel oil–water separation material, FXBW-PU, was developed, with a water contact angle of 155.3°. The FXBW-PU sponge exhibited an absorption capacity ranging from 11.79 to 26.59 g/g for various oils and organic solvents, while maintaining an excellent selective adsorption performance, even after undergoing ten compression cycles, due to its exceptional chemical and mechanical stability. With the assistance of a vacuum pump, the FXBW-PU sponge was utilized in a continuous separation apparatus, resulting in a separation efficiency exceeding 98.6% for various oils and organic solvents. The separation efficiency of n-hexane remains as high as 99.2% even after 10 consecutive separation cycles. Notably, the FXBW-PU sponge also separated the dichloromethane-in-water emulsions, which achieved the effect of purifying water. In summary, FXBW-PU sponge has great potential in the field of cleaning up oil/organic solvent contamination due to its low preparation cost, environmental friendliness and excellent performance.

## 1. Introduction

With the advancement of industrialization, frequent oil spills and large-scale chemical discharges have resulted in severe water pollution. This not only causes significant ecological damage to the global environment, but also poses a grave risk to human health [1,2,3,4,5]. Therefore, it is an urgent and challenging task to deal with oil spills and chemical agents in water. Many methods are currently used to solve this problem, such as biological treatments [6], oil skimming [7], gravity separation [8], in situ combustion [9], etc. Although these methods have certain effects, most of them are energy and time consuming, and are prone to causing secondary pollution in the environment. In addition, adsorption is considered to be a more promising approach because it does not cause adverse environmental effects when treating oil spills and organic solvents in water [10,11,12]. Commonly used adsorbents include graphite, zeolite, cellulose, activated carbon, etc. [13,14,15], but their application for oil–water separation is limited by their poor adsorption capacity, poor recoverability and their inability to be recycled. Therefore, a new adsorbent material with high selectivity, high adsorption capacity and excellent recyclability is urgently needed for the more effective adsorption of oil spills and harmful organic solvents in water.

In recent years, multifunctional materials with special wettability have become a hot research area for oil–water separation applications because of their effective selective absorption capacity (absorbing oil and organic solvents but completely rejecting water) [16]. In addition, the large surface areas and strong adsorption capacities of three-dimensional porous materials are considered ideal for the preparation of special infiltrative materials for oil–water separation [17,18,19]. Among them, polyurethane sponge (PUS) is popular because of its high void ratio, light weight, high adsorption capacity, strong elasticity and other advantages. However, PUS cannot be used directly due to its simultaneous absorption of oil and water. To date, there has been a large body of literature reporting the successful adsorption of oil and organic matter from water by altering the wettability of the polyurethane sponge surface [20,21,22]. Huang et al. [23] prepared superhydrophobic polyurethane sponge with a hierarchical structure by copolymerizing dopamine and n-dodecyl mercaptan using a one-step copolymerization method, which resulted in excellent hydrophobic and oleophilic properties and an adsorption capacity of 24.94–86.70 g/g. Liu et al. [24] developed superhydrophobic sponge by generating roughly structured PDA nanoaggregates on the PUS backbone under alkaline conditions, after being modified by hexamethyldisilazane (HMDS); this had a good selective adsorption ability, and showed good elasticity and recovery ability in compression tests. Tian et al. [25] modified laminated double hydroxide (LDH) sheets with polymethyl hydrogen silicone oil (PMHS) to make them superhydrophobic, and after fixing these superhydrophobic LDH sheets onto polyurethane (PU) using a silane coupling agent (KH550), the derived superhydrophobic sponge had a good oil storage performance and reusability. Great progress has been made in the development oil–water separation techniques by previous authors, but the rigid conditions of the process of hydrophobic modification, the complexity of this process and the high cost of the chemical reagents have prevented mass production [26,27,28]. Even long-chain fluorocarbon materials are non-biodegradable and contaminating to the environment, as well as potentially toxic to humans [29]. Therefore a simple, low-cost preparation method for polyurethane superhydrophobic sponge that can have oil–water separation applications is essential.

Silicone rubber is widely used in the preparation of superhydrophobic materials because of its good mechanical properties and low surface energy characteristics [30,31,32,33]. Silicone rubber composite insulator is widely used in the electrical power industry [34]. As the natural decomposition of silicone rubber is extremely difficult, discarded composite insulator seriously pollutes the environment. How to reasonably recycle waste silicone rubber composite insulators has become an urgent problem in the electrical industry today. In this study, we prepared a powder with good superhydrophobic properties using the umbrella skirt of waste silicone rubber composite insulators. This powder was used to create a superhydrophobic polyurethane sponge that can separate oil and water.

In this paper, a kind of superhydrophobic and oleophilic polyurethane sponge was prepared for oil–water separation. In order to verify its effectiveness and environmental stability, we conducted a number of performance tests. The absorption capacity and cyclic absorption energy of FXBW-PU sponge for various oils and organic solvents were investigated. The continuous separation efficiency was studied using a self-designed oil–water separation device. The research results can be used to purify oil/organic solvent contamination in water.

## 2. Experimental Section

### 2.1. Materials

Polyurethane sponges are very common and can be purchased from local supermarkets. The sponges were washed with anhydrous ethanol and distilled water, and then dried. Silicone rubber composite insulators were supplied by Hubei Electric Power Co., Ltd. (Wuhan, China). Polydimethylsiloxane (PDMS) and curing agent (sylgard184) were supplied by Dow Corning Co., Ltd. (Shanghai, China). Hexane, anhydrous ethanol and ethyl acetate were supplied by Sinopharm Chemical Reagent Co., Ltd. (Shanghai, China). In addition, all other chemicals used were analytical grade reagents and were used in strict accordance with the received standards.

### 2.2. Preparation of FXBW-PU Sponge

#### 2.2.1. Preparation of FXBW Powder

**Figure 1:**
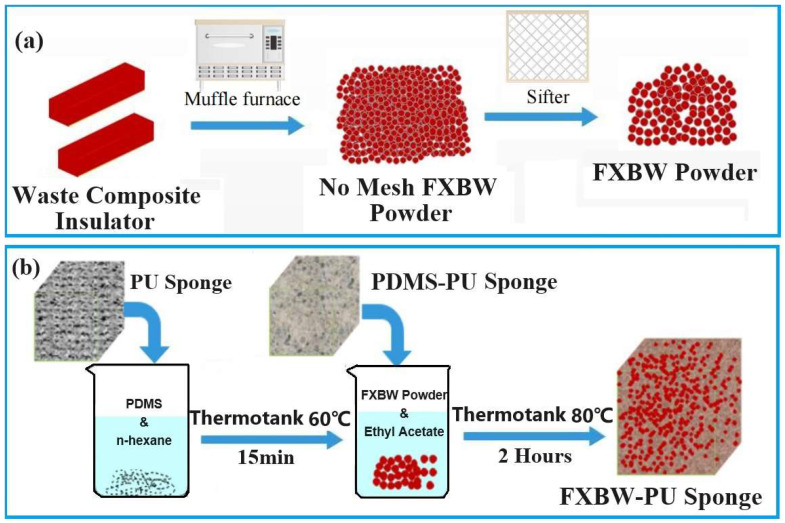
Schematic illustration of the preparation of (**a**) FXBW powder and (**b**) FXBW-PU sponge.

The umbrella skirts of silicone rubber composite insulators were cut into pieces, washed with anhydrous ethanol and deionized water in sequence, and finally put into the drying oven for drying. The dried samples were put into the muffle furnace and burned at 180 °C for 20 min. Subsequently, the burned materials were placed into a mortar and pestled and ground into powder. Lastly, the FXBW powder was obtained by sifting the powder with a size 80 mesh sifter. The whole preparation process is shown in Figure 1a.

#### 2.2.2. Preparation of FXBW-PU Sponge

PDMS is a special organic polymer with a certain hydrophobicity, and has a certain viscosity when it is mixed with a curing agent. It is often used for preparing hydrophobic products [35,36]. Here, 0.5 g PDMS and 0.05 g curing agent—which were used to fix the powder in the sponge space—were dissolved in 15 mL hexane solution and the mixed solution was stirred for 30 min by a magnetic stirrer. The sponge (2 cm × 2 cm × 1 cm) was immersed in the solution for 10 min and then dried in a drying oven at 60 °C for 15 min to evaporate the hexane. Then, different amounts of screened powder (1, 1.5, 2, 2.5, 3 g) were added and poured into 10 g of ethyl acetate solution. The mixture was sonicated at 1 KHz for 30 min and magnetically stirred for 30 min. Finally, the dried FXBW-PU sponge was immersed in the above solution, removed after 10 min and dried under a vacuum at 80 °C for 2 h to obtain the superhydrophobic sponge (FXBW-PU), as shown in Figure 1b.

### 2.3. Characterization

Both the static water contact angle and the sliding angle were measured at room temperature with a measuring instrument (JY-PHB, produced by Hebei Chengde Jinhe Instrument Factory, Shijiazhuang, China). The morphology of the prepared sponge surface was obtained via field emission scanning electron microscopy (SEM, JSM-7500F, Tokyo, Japan). The elemental distribution of the samples was obtained using energy spectrometry (EDS, Kevex, Thermo Scientific, Waltham, MA, USA). Functional group spectra were recorded with a Fourier transform infrared spectrometer (FTIR, Nexus 870, Thermo Scientific, Waltham, MA, USA). The morphology of the emulsions before and after separation was observed by optical microscopy (Primotech, Zeiss, Oberkochen, Germany).

### 2.4. Adsorption Test

To evaluate the maximum adsorption capacity for various solvents and oils, the FXBW-PU sponge specimens were separately immersed in solutions of motor oil, soybean oil, silicone oil, n-hexane, petroleum ether and methylene chloride. The specimens full of various oils were taken out to weigh and then all liquids were squeezed into measuring cups. We measured the water content α_1_ of the liquids with a Cassell moisture analyzer made by Metrohm. The test was repeated three times and the absorption capacity Q was formulated as follows:

(1) $$Q(g/g)=\frac{{(1-\alpha_{1})m}_{sorp}-m}{m}$$

where $m_{sorp}$ is the mass of the sponge after oil absorption and *m* is the mass of the sponge before oil absorption.

The circulation adsorption capacity of the sponge was tested using the following method. Tweezers were used to squeeze the absorbed oil sponges until the oil was no longer dripping out, and then the sponges were washed three times with ethanol and dried in a drying oven at 60 °C. Lastly, the maximum adsorption capacity was measured again.

### 2.5. Continuous Oil–Water Separation Test

A simple oil–water separation device, composed of a vacuum pump, a rubber tube, a filter extraction bottle and the FXBW-PU sponge, was built. The oil in the oil–water mixture entered the extraction bottle along the rubber tube through the FXBW-PU sponge via the vacuum pump. Then, we measured the water content α_2_ of the liquids in the bottles with the Cassell moisture analyzer. Considering the possible residual oil in the rubber hose in the separation device, its separation efficiency *E* was calculated using the following formula:

(2) $$E=\frac{\left(1-\alpha_{2}\right)(m_{1}-m_{2})}{m_{0}}\times 100\%$$

where $m_{1}$ and $m_{2}$ are the masses of the oil–water mixture before and after separation, respectively, and $m_{0}$ is the net weight of the oil in the mixture.

### 2.6. Oil-in-Water Emulsion Preparation

Referring to a previous report on the preparation of oil-in-water emulsions [37], a mixture containing dichloromethane–water solution (*v*/*v*, 1: 50) was first prepared by ultrasonication for 30 min and then stirred with a magnetic stirrer for 30 min to form a stable oil-in-water emulsion.

## 3. Results and Discussion

### 3.1. Characterization of FXBW-PU Sponge

**Figure 2:**
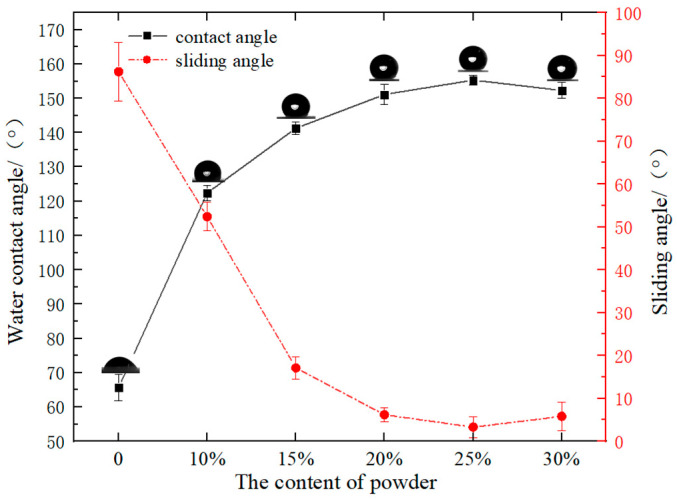
The change curves of hydrophobicity of FXBW-PU sponge with the content of FXBW powder.

Two critical factors for preparing superhydrophobic materials are micro- and nanostructures and a low surface energy [38]. The burned scrap silicone rubber composite insulator (FXBW) powder itself is superhydrophobic, providing a micro- and nanostructure and a low surface energy. The superhydrophobic sponge was prepared by fixing the FXBW powder onto the polyurethane sponge. To enhance the binding force of the FBXW powder and the sponge, PDMS adhesives were introduced. To investigate the effect of the powder content on the hydrophobicity of the prepared sponge, the wettability change curves of the sponges prepared in five different solution concentrations ratios were tested, as shown in Figure 2. It is clearly shown that with the increase of powder content, the water contact angle (WCA) of the sponge increased initially, and then decreased, while the water sliding angle (WSA) decreased initially, and then increased. The WCA of the FXBW-PU sponge surface reached the maximum value of 155.3° to achieve superhydrophobicity when the concentration of FXBW powder was 25%.

**Figure 3:**
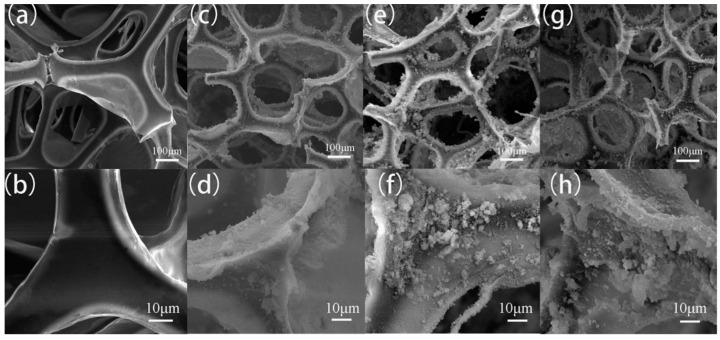
SEM images of FXBW-PU sponges with different powder contents: (**a**,**b**) original (**c**,**d**) 15%; (**e**,**f**) 25%; (**g**,**h**) 30%.

The surface microtopography of FXBW-PU sponges prepared with different powder contents is shown in Figure 3. The surface of the original sponge was very smooth, basically without any powder deposition. As the concentration of the FXBW powder solution increased, the skeleton of the polyurethane sponge showed powder deposition and became rougher and rougher. When the concentration ratio of the FXBW powder solution reached 30%, the excessive FXBW powder began to block the holes within the polyurethane sponge skeleton and destroyed its three-dimensional porous structure, which may reduce the adsorption capacity and superhydrophobicity of the sponge. It is obvious that the superhydrophobicity of the prepared FXBW-PU sponge was optimal when the concentration ratio of the FXBW powder solution was 25%. In the following experiments, all of the FXBW-PU sponges were prepared with an FXBW powder solution concentration of 25%.

### 3.2. Wettability of FXBW-PU Sponge

**Figure 4:**
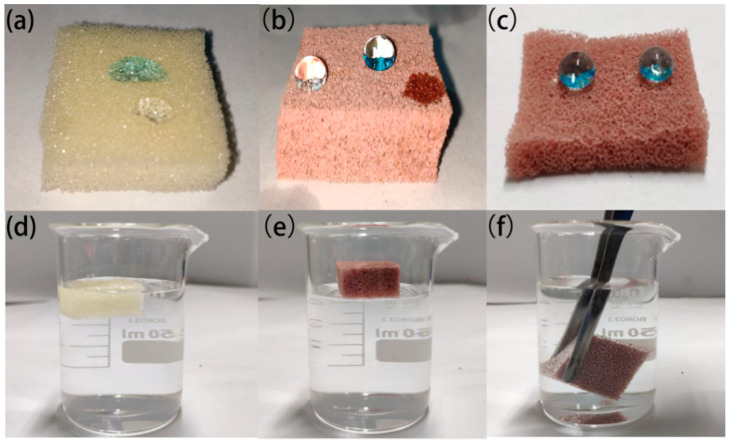
(**a**) Water drops on the original sponge (the blue color is methylene blue staining); (**b**) water drops and oil on FXBW-PU sponge; (**c**) water drops on the internal section of FXBW-PU sponge. (**d**) Original sponge; and (**e**) FXBW-PU sponge in water, respectively. (**f**) The silver mirror phenomenon of FXBW-PU sponge.

Here, we tested the water and the oil wettability of the untreated sponge and the FXBW-PU sponge. In Figure 4a, water droplets were spread and soaked into the surface of the original sponge, showing its hydrophilicity. Corresponding to Figure 4b, it w clear that the water droplets were quasi-spherical and stable on the surface of the FXBW-PU sponge, but that the oil was absorbed by the FXBW-PU sponge immediately. The FXBW-PU sponge has good oleophilic and superhydrophobic properties, which provides the basis for the separation of oil and water later. Figure 4c shows a photograph of water droplets on an internal, arbitrary cross-section of the FXBW-PU sponge, which shows that the interior of the FXBW-PU sponge is also superhydrophobic. The high solubility of ethyl acetate promoted the solution, with FXBW powder flowing into the sponge interior [39]. As shown in Figure 4d,e, the original sponge and the FXBW-PU sponge were placed in water. As the original polyurethane sponge itself has a three-dimensional porous structure and hydrophilic properties, it absorbed enough water and sank into the water. The FXBW-PU sponge floated on the water. When immersing the FXBW-PU sponge in water with external force, we observed the silver mirror phenomenon on the surface of the sponge, as shown in Figure 4f. The reason for this is that the interior of the FXBW-PU sponge is filled with air, which hinders the infiltration of water and shows that FXBW-PU sponge has good hydrophobic properties.

**Figure 5:**
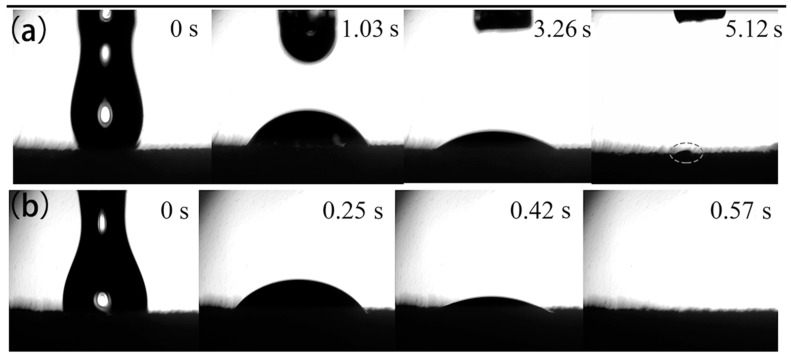
Absorption process of soybean oil by (**a**) original sponge and (**b**) FXBW-PU sponge.

Figure 5 shows the process of soybean oil being absorbed by the original sponge and the FXBW-PU sponge. Figure 5a shows that it took 5.12 s for 6 ul of soybean oil to be basically absorbed by the original sponge, but as there was still a little soybean oil, there was not complete absorption. In contrast, in Figure 5b, soybean oil was completely absorbed by the FXBW-PU sponge after only 0.57 s, which demonstrates that the modified sponge has super oleophilic properties. 

### 3.3. Stability of FXBW-PU Sponge

**Figure 6:**
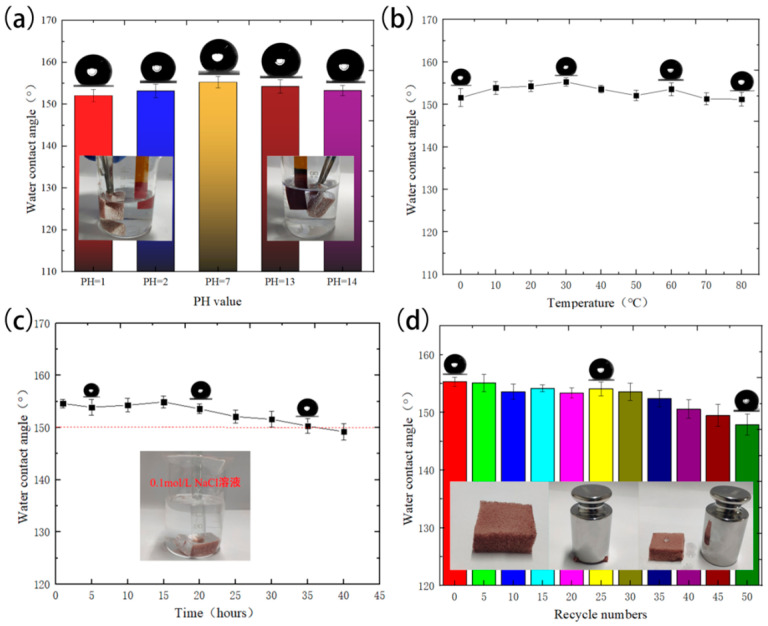
Change curves of the water contact angle of FXBW-PU sponge after undergoing different stability tests: (**a**) acid-base corrosion; (**b**) temperature; (**c**) NaCl solution; and (**d**) recycle.

The leakage of petroleum and organic solvents usually occurs in very complex environments, so it is essential to study the corrosion resistance, high-temperature resistance and durability of materials. To investigate its corrosion resistance, the FXBW-PU sponge was immersed in solutions with different pH values for 24 h and then cleaned with deionized water. The WCAs of the FXBW-PU sponges were measured after being dried in a drying oven, as shown in Figure 6a; the WCAs were still higher than 150° after impregnation with different PH solutions, which indicates that FXBW-PU sponge has good corrosion resistance. In addition, the variation of the WCA of the FXBW-PU sponge which was impregnated in 1 mol/L NaCl solution is shown in Figure 6c. The FXBW-PU sponge still maintained its superhydrophobicity after 35 h of continuous soaking. To investigate thermal stability performance, the FXBW-PU sponges were placed in a constant temperature chamber (0–80 °C) at different temperatures for 2 h and then the WCAs were measured. As Figure 6b shows, all of the WCAs were more than 150° when the temperature was varied from 0 to 80 °C. In addition, a 200g weight was loaded onto each FXBW-PU sponge and maintained for 1 min, which induced deformation. Then, the weight was removed and the WCAs were measured. The variation of the WCAs with compression cycles is shown in Figure 6f. As can be seen from the graph, the WCA of the FXBW-PU sponge after 40 compression cycles was still greater than 150°; after more than 40 compression cycles, the hydrophobicity of the FXBW-PU sponge reduces. All the above tests demonstrate that the FXBW-PU sponge has a good stability performance.

### 3.4. Oil–Water Separation Function of FXBW-PU Sponge

**Figure 7:**
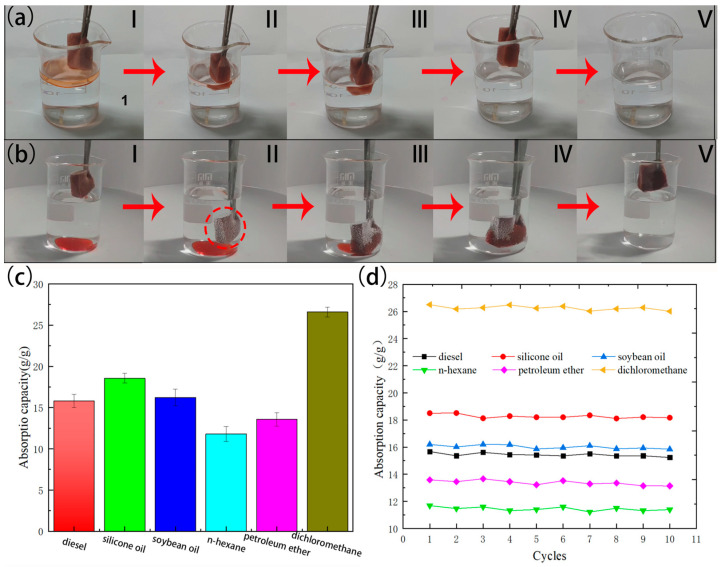
The process diagram of FXBW-PU sponge absorbing (**a**) hexane (Sudan III dyeing) floating on the water and (**b**) dichloroethylene chloride (Sudan III dyeing) underwater from I to V; (**c**) the one-time maximum adsorption capacity of FXBW-PU sponge for various oils and organic solvents; and (**d**) the change curves of the adsorption capacity of FXBW-PU sponge for different oils and organic solvents with the number of cycles.

The superhydrophobicity and superoleophilicity of the FXBW-PU sponge can be used to efficiently absorb oil and organic solvents from water. Generally, due to their different densities within water, oil or organic pollutants will float on the surface or sink to the bottom of the water. In order to simulate the process of absorbing oil, due to their different densities, hexane and methylene chloride (both dyed red using Sudan III) were selected as they are a light oil and a heavy oil, respectively. Figure 7a and Video S1 show the absorption process of n-hexane by the FXBW-PU sponge. It can be seen that the hexane floating on the water surface was quickly absorbed by the FXBW-PU sponge, leaving behind clear and transparent water. In addition, Figure 7b and Video S2 illustrate the process of dichloromethane uptake by the FXBW-PU sponge. The FXBW-PU sponge was immersed in a solution of dichloromethane and water. The surface exhibited the “silver mirror phenomenon” when the sponge was in close proximity to the dichloromethane. Due to the superhydrophobic properties of the FXBW-PU sponge, the dichloromethane rapidly absorbed into the sponge without leaving any residue on its surface when removed from the water. The aforementioned tests demonstrate the commendable hydrophobic and oleophilic properties of FXBW-PU sponge.

Maximum adsorption capacity is a crucial parameter for evaluating the absorption performance of various oils and organic solvents. The absorption capacity of FXBW-PU sponge was assessed for six oils and organic solvents, including motor oil, soybean oil, silicone oil, n-hexane, petroleum ether and methylene chloride. The specific absorption capacity is illustrated in Figure 7c. The oil adsorption capacity of FXBW-PU sponge containing a trace water content of about 1.57%~2.32% ranged from 11.79 to 26.59 g/g, which was generally positively correlated with the density of the oil or organic solvent. The internal volume of the FXBW-PU sponge remained constant; thus, its absorption capacity for a given volume increases with the density of oil or organic solvent [40].

Reusability is a crucial criterion for evaluating the efficacy of superhydrophobic sponges in oil–water separation. As depicted in Figure 7d, the maximum adsorption capacity of FXBW-PU sponge for six oils and organic solvents exhibited only a marginal decrease after ten absorption cycles. The sponge demonstrated a reduction in adsorption capacity of 2.15% and 4.08%, respectively, relative to its initial adsorption capacity across various solvents. The solvent recovery method was accomplished by compressing the FXBW-PU sponge. The FXBW-PU sponge’s oil storage capacity may be reduced due to irreversible deformation caused by the packing density, but its adsorption performance remains favorable owing to its inherent structure.

**Figure 8:**
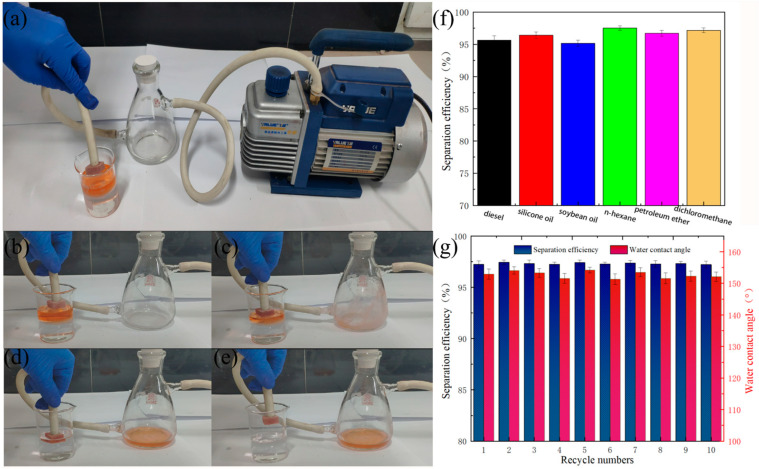
(**a**–**e**) The process of separating n-hexane (Sudan Red III dyed) from water using FXBW-PU sponge; (**f**) the separation efficiency of FXBW-PU sponge for various oils and organic solvents; and (**g**) the variation of water contact angle and the separation efficiency for n-hexane with the number of cycles.

In the practical application process, if the absorption–extrusion cycle is found to be inefficient for oil–water separation, a vacuum pump-assisted FXBW-PU sponge with constant oil absorption can achieve the rapid and continuous separation of oil and water. Figure 8a–e and Video S3 show the process of separating a mixture of hexane (Sudan III dye) and water using this homemade absorption device. It can be seen that when the FXBW-PU sponge was immersed in the oil–water mixture (*v*/*v* 3:5), under the action of the vacuum pump, the hexane was quickly absorbed into the filter bottle. There were no hexane residues in the beaker and almost no water in the filter bottle, which indicates that the FXBW-PU sponge has a good continuous oil–water separation ability. Figure 8f shows the separation efficiency of the FXBW-PU sponge for six different oils and organic solvents. Among them, the FXBW-PU sponge had the highest separation efficiency, 97.5%, for n-hexane and over 95.4% for other oils. In addition, the separation efficiency and water contact angle of FXBW-PU sponge for hexane–water mixture solutions were also investigated, as shown in Figure 8g along with the number of cycles. It is clear that after 10 cycles, the separation efficiency of the FXBW-PU sponge for hexane had no obvious change—still above 97.2%—and the water contact angle was still higher than 150°. The excellent adhesion ability of PDMS provided a strong bond between the FXBW powder and the polyurethane sponge skeleton, which can slow excessive peeling-off after several successive separations.

### 3.5. Purification Performance of FXBW-PU Sponge on Oil-In-Water Emulsion

**Figure 9:**
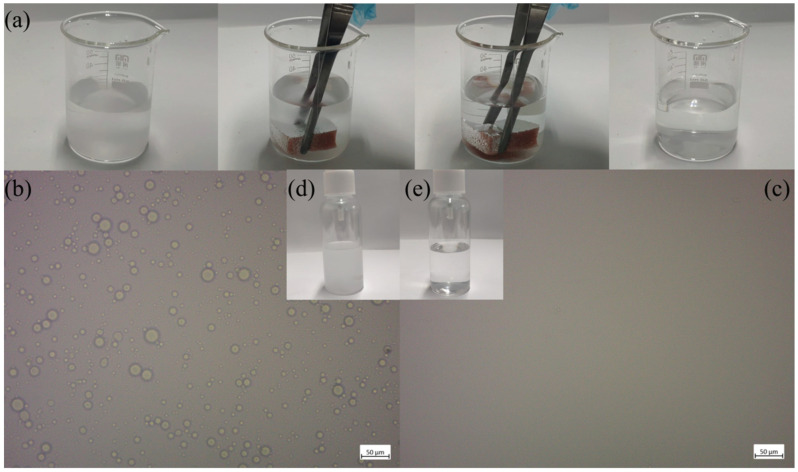
(**a**) The process of purification of oil-in-water using FXBW-PU sponge; the optical microscope pictures (**b**) before and (**c**) after the emulsion purification; and the photos (**d**) before and (**e**) after the oil-in-water emulsion purification.

In practical applications, oil may not only coalesce in water, but may also disperse to form an emulsion, posing a greater challenge for oil–water separation. To evaluate the purification efficiency of FXBW-PU sponge with mixed emulsions, a dichloromethane and water (*v*/*v* 1/50) oil-in-water emulsion was selected as the target pollutant for adsorption experiments. The adsorption process is illustrated in Figure 9a and Video S4. At the beginning of the separation process, an emulsion was present in the beaker and an FXBW-PU sponge was immersed into it. The emulsion gradually attained transparency and was purified within approximately one minute. The changes in emulsion morphology before and after purification were examined using optical microscopy, as illustrated in Figure 9b,c. After purification, the original emulsion, which contained numerous dichloromethane droplets ranging from 3 to 8 µm dispersed in water, exhibited a significant reduction in micron-sized droplets within the solution. The above phenomenon serves as evidence that the FXBW-PU sponge possesses the ability to effectively purify oil emulsions, thereby rendering it suitable for employment in more intricate oil spill remediation and recovery efforts.

## 4. Conclusions

In this study, a specialized polyurethane sponge with unique wettability was fabricated through a straightforward solution impregnation method that fixed silicone rubber powder onto the skeleton of the sponge. The implementation is facile and eco-friendly, with the added benefit of recycling utilized silicone rubber insulators. The prepared FXBW-PU sponge exhibited remarkable superhydrophobic and oleophilic properties, as well as excellent mechanical and chemical stability. The product demonstrates a discerning affinity for oil slicks on water and heavy oils submerged underwater. The FXBW-PU sponge showcased an adsorption capacity ranging from 11.79 to 26.59 times its own weight for various oils and organic solvents, while maintaining exceptional performance even after undergoing ten compression cycles. With the aid of a vacuum pump, oil and organic matter can be continuously and rapidly recovered with a separation efficiency exceeding 98.6%. Additionally, the FXBW-PU sponge exhibited efficacy in removing oil droplets from oil-in-water emulsions, indicating its potential for cleaning oil/organic solvent contamination.

## Supplementary Materials

The following supporting information can be downloaded at: https://www.mdpi.com/article/10.3390/ma16186320/s1, Video S1: The process of FXBW-PU sponge absorbing hexane; Video S2: the process of FXBW-PU sponge absorbing dichloroethylene chloride; Video S3: the process of separating a mixture of hexane (Sudan III dye) and water using the homemade absorption device. Video S4: the process of purification of oil-in-water using FXBW-PU sponge.

## References

[1] Singh H., Bhardwaj N., Arya S.K., Khatri M. (2020). Environmental impacts of oil spills and their remediation by magnetic nanomaterials. Environ. Nanotechnol. Monit. Manag..

[2] Ren H., Hao J., Kang W., Wang G., Ju J., Li L., Cheng B. (2019). Waste spunlaced facial puff derived monolithic flexible carbon framework (WCF): An ultralow-cost, recyclable and eco-friendly sorbent for oils and organic solvents. RSC Adv..

[3] Zhou Y., Gu X., Yuan Z., Li Y., Wang B., Yan J., Zhao D., Liu J., Liu X. (2022). PDMS mesh with reversible super-wettability for oil/water separation. Colloids Surf. A Physicochem. Eng. Asp..

[4] Qiu L., Sun Y., Guo Z. (2020). Designing novel superwetting surfaces for high-efficiency oil–water separation: Design principles, opportunities, trends and challenges. J. Mater. Chem. A.

[5] Ren G., Song Y., Li X., Zhou Y., Zhang Z., Zhu X. (2018). A superhydrophobic copper mesh as an advanced platform for oil-water separation. Appl. Surf. Sci..

[6] Chuah L.F., Chew K.W., Bokhari A., Mubashir M., Show P.L. (2022). Biodegradation of crude oil in seawater by using a consortium of symbiotic bacteria. Environ. Res..

[7] Visco A., Quattrocchi A., Nocita D., Montanini R., Pistone A. (2021). Polyurethane foams loaded with carbon nanofibers for oil spill recovery: Mechanical properties under fatigue conditions and selective absorption in oil/water mixtures. Nanomaterials.

[8] Liao X., Li H., Su X., Zhan H., Lai X., Zeng X. (2019). Mussel-inspired cotton fabric with pH-responsive superwettability for bidirectional oil–water separation. J. Mater. Sci..

[9] Rojas-Alva U., Andersen B.S., Jomaas G. (2019). Chemical herding of weathered crude oils for in-situ burning. J. Environ. Manag..

[10] Yue X., Li Z., Zhang T., Yang D., Qiu F. (2019). Design and fabrication of superwetting fiber-based membranes for oil/water separation applications. Chem. Eng. J..

[11] Kang H., Zhao B., Li L., Zhang J. (2019). Durable superhydrophobic glass wool@polydopamine@PDMS for highly efficient oil/water separation. J. Colloid Interface Sci..

[12] Kukkar D., Rani A., Kumar V., AliYounis S., Zhang M., Lee S.-S., Tsang D.C.W., Kim K.-H. (2020). Recent advances in carbon nanotube sponge–based sorption technologies for mitigation of marine oil spills. J. Colloid Interface Sci..

[13] Fürtauer S., Hassan M., Elsherbiny A., Gabal S.A., Mehanny S., Abushammala H. (2021). Current status of cellulosic and nanocellulosic materials for oil spill cleanup. Polymers.

[14] Zamparas M., Tzivras D., Dracopoulos V., Ioannides T. (2020). Application of sorbents for oil spill cleanup focusing on natural-based modified materials: A review. Molecules.

[15] Yang J., Wang H., Tao Z., Liu X., Wang Z., Yue R., Cui Z. (2019). 3D superhydrophobic sponge with a novel compression strategy for effective water-in-oil emulsion separation and its separation mechanism. Chem. Eng. J..

[16] Toro R.G., Calandra P., Federici F., de Caro T., Mezzi A., Cortese B., Pellegrino A.L., Malandrino G., Caschera D. (2020). Development of superhydrophobic, self-cleaning, and flame-resistant DLC/TiO_2_ melamine sponge for application in oil–water separation. J. Mater. Sci..

[17] Wang Y., Feng Y., Yao J. (2019). Construction of hydrophobic alginate-based foams induced by zirconium ions for oil and organic solvent cleanup. J. Colloid Interface Sci..

[18] Wang M., Zhu J., Zi Y., Huang W. (2021). 3D MXene sponge: Facile synthesis, excellent hydrophobicity, and high photothermal efficiency for waste oil collection and purification. ACS Appl. Mater. Interfaces.

[19] Dashairya L., Sahu A., Saha P. (2019). Stearic acid treated polypyrrole-encapsulated melamine formaldehyde superhydrophobic sponge for oil recovery. Adv. Compos. Hybrid Mater..

[20] Cheng Z., Li J., Wang B., Zeng J., Xu J., Gao W., Zhu S., Hu F., Dong J., Chen K. (2020). Scalable and robust bacterial cellulose carbon aerogels as reusable absorbents for high-efficiency oil/water separation. ACS Appl. Bio Mater..

[21] Wang X., Pan Y., Liu X., Liu H., Li N., Liu C., Schubert D.W., Shen C. (2019). Facile fabrication of superhydrophobic and eco-friendly poly (lactic acid) foam for oil–water separation via skin peeling. ACS Appl. Mater. Interfaces.

[22] Seeharaj P., Pasupong P., Detsri E., Damrongsak P. (2018). Superhydrophobilization of SiO_2_ surface with two alkylsilanes for an application in oil/water separation. J. Mater. Sci..

[23] Zhang J., Zhao J., Qu W., Li X., Wang Z. (2020). One-step, low-cost, mussel-inspired green method to prepare superhydrophobic nanostructured surfaces having durability, efficiency, and wide applicability. J. Colloid Interface Sci..

[24] Liu C., Fang Y., Miao X., Pei Y., Yan Y., Xiao W., Wu L. (2019). Facile fabrication of superhydrophobic polyurethane sponge towards oil-water separation with exceptional flame-retardant performance. Sep. Purif. Technol..

[25] Tian Q., Liu Q., Zhou J., Ju P., Waterhouse G.I., Zhou S., Ai S. (2019). Superhydrophobic sponge containing silicone oil-modified layered double hydroxide sheets for rapid oil-water separations. Colloids Surf. A Physicochem. Eng. Asp..

[26] Huang S., Zhang Y., Shi J., Huang W. (2016). Superhydrophobic particles derived from nature-inspired polyphenol chemistry for liquid marble formation and oil spills treatment. ACS Sustain. Chem. Eng..

[27] Lei Z., Zhang G., Deng Y., Wang C. (2017). Surface modification of melamine sponges for pH-responsive oil absorption and desorption. Appl. Surf. Sci..

[28] Dashairya L., Gopinath M., Saha P. (2020). Synergistic effect of Zr/Cl dual-ions mediated pyrrole polymerization and development of superhydrophobic melamine sponges for oil/water separation. Colloids Surf. A Physicochem. Eng. Asp..

[29] Jiang C., Liu W., Yang M., Liu C., He S., Xie Y., Wang Z. (2018). Facile fabrication of robust fluorine-free self-cleaning cotton textiles with superhydrophobicity, photocatalytic activity, and UV durability. Colloids Surf. A Physicochem. Eng. Asp..

[30] Wang G., Li A., Li K., Zhao Y., Ma Y., He Q. (2021). A fluorine-free superhydrophobic silicone rubber surface has excellent self-cleaning and bouncing properties. J. Colloid Interface Sci..

[31] Li A., Wang G., Ma Y., Zhao C., Zhang F., He Q., Zhang F. (2021). Study on preparation and properties of superhydrophobic surface of RTV silicone rubber. J. Mater. Res. Technol..

[32] Zhang M., Guo C., Hu J. (2020). One-step fabrication of flexible superhydrophobic surfaces to enhance water repellency. Surf. Coatings Technol..

[33] Hu Y., Ma X., Bi H., Sun J. (2020). Robust superhydrophobic surfaces fabricated by self-growth of TiO2 particles on cured silicone rubber. Colloids Surf. A Physicochem. Eng. Asp..

[34] Yan H., Dai X., Ruan K., Zhang S., Shi X., Guo Y., Cai H., Gu J. (2021). Flexible thermally conductive and electrically insulating silicone rubber composite films with BNNS@Al_2_O_3_ fillers. Adv. Compos. Hybrid Mater..

[35] Sulym I., Goncharuk O., Sternik D. (2017). Nanooxide/Polymer Composites with Silica@PDMS and Ceria–Zirconia–Silica@PDMS: Textural, Morphological, and Hydrophilic/Hydrophobic Features. Nanoscale Res. Lett..

[36] Lai C.C., Chung C.K. (2020). Hydrophilicity and optic property of polyethylene glycol coating on polydimethylsiloxane for fast prototyping and its application to backlight microfluidic chip. Surf. Coat. Technol. Surf. Coat. Technol..

[37] Zhou Q., Chen G., Xing T. (2018). Facile construction of robust superhydrophobic tea polyphenol/Fe@cotton fabric for self-cleaning and efficient oil–water separation. Cellulose.

[38] Guo Z., Liu W., Su B.-L. (2011). Superhydrophobic surfaces: From natural to biomimetic to functional. J. Colloid Interface Sci..

[39] Guan H., Cheng Z., Wang X. (2018). Highly compressible wood sponges with a spring-like lamellar structure as effective and reusable oil absorbents. ACS Nano.

[40] Gong X., Wang Y., Zeng H., Betti M., Chen L. (2019). Highly porous, hydrophobic, and compressible cellulose nanocrystals/poly (vinyl alcohol) aerogels as recyclable absorbents for oil–water separation. ACS Sustain. Chem. Eng..

